# Convergent validity of the EQ-5D-3L in a randomized-controlled trial of the Housing First model

**DOI:** 10.1186/s12913-019-4310-z

**Published:** 2019-07-12

**Authors:** Nicole Kozloff, Andrew D. Pinto, Vicky Stergiopoulos, Stephen W. Hwang, Patricia O’Campo, Ahmed M. Bayoumi

**Affiliations:** 1 Centre for Addiction and Mental Health, 250 College Street, 7th floor, Toronto, ON M5T 1R8 Canada; 2 Centre for Urban Health Solutions, Li Ka Shing Knowledge Institute, St. Michael’s Hospital, 30 Bond Street, Toronto, ON M5B1W8 Canada; 3 Department of Psychiatry, University of Toronto, 250 College Street, Toronto, ON M5T1R8 Canada; 4 Institute of Health Policy, Management, and Evaluation, University of Toronto, Health Sciences Building, 155 College Street, Suite 425, Toronto, ON M5T 3M6 Canada; 5 Department of Family and Community Medicine, St. Michael’s Hospital, 30 Bond Street, Toronto, ON M5B 1W8 Canada; 6 Department of Family and Community Medicine, Faculty of Medicine, University of Toronto, 500 University Avenue, 5th Floor, Toronto, ON M5G 1V7 Canada; 7 Dalla Lana School of Public Health, University of Toronto, Health Sciences Building, 155 College Street, 6th Floor, Toronto, ON M5T 3M7 Canada; 8 Department of Medicine, University of Toronto, Suite RFE 3-805, 200 Elizabeth Street, Toronto, ON M5G 2C4 Canada; 9 Division of General Internal Medicine, St. Michael’s Hospital, 30 Bond Street, Toronto, ON M5B 1W8 Canada

**Keywords:** Vulnerable populations/access to care, Psychiatry, Utility measurement, EQ-5D, QOL in special populations

## Abstract

**Background:**

Health utility assessments are important for economic evaluations but few instruments have been validated in homeless people with mental illness. We examined the convergent validity of the EuroQol-5 Dimension 3-level questionnaire (EQ-5D-3L) as a measure of quality of life in homeless adults with mental illness.

**Methods:**

Data were from Toronto participants in At Home/Chez Soi, a 24-month randomized controlled trial of Housing First (immediate access to scattered site housing and mental health support services) compared to treatment as usual for homeless adults with a mental disorder (*n* = 575). Participants completed the EQ-5D-3L at 6 month intervals. We tested convergent validity, hypothesizing strong correlation (*r* > 0.6) with the Lehman Quality of Life Interview 20 (QOLI-20) index and moderate correlations (*r* > 0.3) with the Colorado Symptom Index (CSI), Recovery Assessment Scale (RAS), and number of comorbidities. We also examined correlations between EQ-5D-3L scores and the QOLI-20 over time using a linear mixed-effects model.

**Results:**

The EQ-5D-3L was not strongly correlated with the QOLI-20 (r ranged from 0.31–0.52 at various time points). The EQ-5D-3L was moderately correlated with the CSI, RAS, and number of comorbidities. The Snijders/Bosker r^2^ for longitudinal validity between the EQ-5D-3L and QOLI-20 within subjects over time was 0.2094 (square-root *r* = 0.4576).

**Conclusions:**

The EQ-5D-3L did not demonstrate strong convergent validity in homeless people with mental illness but was moderately correlated with several instruments. Further research is warranted to determine the optimal method for measuring health utilities in this population.

**Trial registration:**

International Standard Randomised Control Trial Registry ISRCTN42520374 assigned on August 18, 2009.

**Electronic supplementary material:**

The online version of this article (10.1186/s12913-019-4310-z) contains supplementary material, which is available to authorized users.

## Background

Homelessness is associated with increased mortality and morbidity relative to the general population, including a high prevalence of mental health and substance use disorders [1]. Evaluating programs to address homelessness requires accurate measurement of the associated health, social, and economic consequences. Because such interventions can be expensive, demonstrating that programs are cost-effective is important for health policy decision makers. Cost-effectiveness analyses in health typically rely on outcome measures such as quality adjusted life years, which integrate survival and quality of life measured using preference-based instruments that produce utility scores [2]. Utilities are anchored at 0 (equivalent to death) and 1 (equivalent to best possible health), although utility scales can also have negative values for health states that are considered worse than death [3]. Utilities can be elicited *directly* from interviews with individuals using instruments such as the standard gamble or time trade-off or estimated *indirectly* from generic health-related quality of life questionnaires, in which individual responses to specific questions are transformed into utility scores based on weights, frequently derived from a sample of community members [4]. Indirect utilities are preferred by some economists who view community ratings as the most appropriate approach to capture preferences for societal-level decision making [5]. They may also require less cognitive effort [6] and cause less distress [7] than direct methods, so may be more acceptable to participants, particularly those with mental illness.

The EuroQol-5 Dimension 3-level questionnaire (EQ-5D-3L) is a popular indirect utility instrument [8]. It has been recommended as the preferred method for measuring health-related quality of life by the UK’s National Institute for Health and Care Excellence (NICE), except in patient populations for which it performs poorly on tests of construct validity and responsiveness [9]. A recent comprehensive report on the use of generic preference-based measures of health in mental health populations concluded that the EQ-5D and 36-Item Short Form Survey (SF-36) (another indirect utility instrument) achieve an adequate level of psychometric performance in patients with depression and, to some extent, in patients with anxiety and personality disorder; however, results were mixed in patients with serious mental illness such as schizophrenia and bipolar disorder and the report concluded that further validation studies were needed [10]. While specific generic health status instruments have been tested in homeless populations [11, 12], there have been few efforts to validate indirect health utility measures. The EQ-5D-3L has previously been administered to people who are homeless although its validity in this population is uncertain. A Swedish study demonstrated lower EQ-5D-3L scores among homeless adults compared to population norms, with significantly lower scores among those sleeping “rough” (in a setting not intended for habitation) and those reporting symptoms of mental illness [13]. A Toronto study examined the EQ-5D-3L in individuals accepted into supportive housing compared with those on a wait list over time [14]. However, neither study examined its convergent validity with other measures of quality of life and health.

**Fig. 1:**
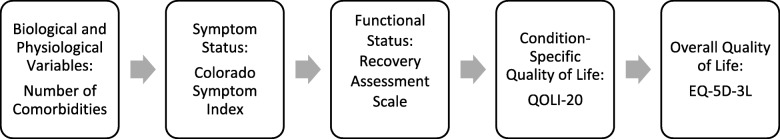
Conceptual model guiding hypotheses about convergent validity of EQ-5D-3L with other measures in At Home/Chez Soi. Based on Wilson and Cleary’s model linking clinical variables with health-related quality of life, we hypothesized that the EuroQol-5 Dimension 3-level questionnaire (EQ-5D-3L) would be strongly correlated with the Lehman Quality of Life Interview 20 (QOLI-20) index and moderately correlated with the Recovery Assessment Scale, Colorado Symptom Index, and number of comorbidities

Convergent validity is a measure of how closely a scale relates to measures of other constructs to which it should be related [15]. One highly-cited conceptual model developed by Wilson and Cleary positions biological and physiological variables, symptom status, functional status, and general health perceptions as being causally associated with overall quality of life [16]. Thus, our objective was to test the convergent validity of the EQ-5D-3L with other health status instruments within the context of the Toronto, Ontario site of the At Home/Chez Soi study, a randomized controlled trial of Housing First in people who are homeless and have mental illness. The Housing First model provided access to scattered-site housing of the individual’s choice through the use of rent supplements and support services without mandating sobriety or psychiatric treatment. We used the Wilson and Cleary model to guide our hypotheses about the relationship between overall quality of life (as measured with the EQ-5D-3L) and other measures in our population (Fig. 1) [16]. We hypothesized that constructs that were more proximal to the EQ-5D-3L according to this conceptual framework should be more strongly correlated than constructs more distal in the model. Specifically, we hypothesized that the EQ-5D-3L would be strongly correlated with the Lehman Quality of Life Interview 20 (QOLI-20) index, a well-established quality of life measure for this population [17], and moderately correlated with the Recovery Assessment Scale [18, 19], Colorado Symptom Index [20, 21], and number of comorbidities. We also examined whether the EQ-5D-3L achieved longitudinal convergent validity by examining correlation over time with the QOLI-20.

## Methods

### Study overview and participants

At Home/Chez Soi trial participants were recruited between 2009 and 2011. Each participant was followed for up to 24 months and the study terminated in 2013. Participants were eligible if they were aged 18 or older, were currently homeless or precariously housed, and had a mental disorder based on Diagnostic and Statistical Manual of Mental Disorders, 4th Edition criteria as determined by the Mini International Neuropsychiatric Interview (MINI) at study entry [22]. Absolute homelessness was defined as having no fixed place to stay for 7 or more nights and little likelihood of obtaining accommodation in the upcoming month; precariously housed was defined as primarily residing in a Single Room Occupancy, rooming house, or hotel/motel, with 2 or more episodes of absolute homelessness in the past year. Individuals were excluded if they were already clients of an assertive community treatment (ACT) or intensive case management (ICM) team, did not have legal status in Canada, or did not meet a strict definition of homelessness. Participants were recruited from community agencies that serve people who are homeless, institutions (e.g., healthcare facilities, prisons and jails), and directly from the street.

All study participants were stratified by level of need. “High needs” individuals: 1) scored below 62 on the Multnomah Community Ability Scale (MCAS) [23, 24], indicating poor community functioning; 2) had a MINI diagnosis of current psychotic or bipolar disorder or psychotic symptoms documented by the interviewer or referring provider at eligibility screening; and 3) had 2 or more hospitalizations for mental illness in any 1 of the 5 years prior to enrolment, comorbid substance use disorder, or answered “yes,” “don’t know,” or declined to answer a question about recent arrest or incarceration. All other eligible participants were classified as “moderate needs.” Patients were randomized according to level of need using adaptive randomization procedures which continually adjust the probability of being assigned to intervention or treatment as usual based on the number of participants already assigned, to increase the likelihood of achieving a balanced number of participants between groups [25].

**Fig. 2:**
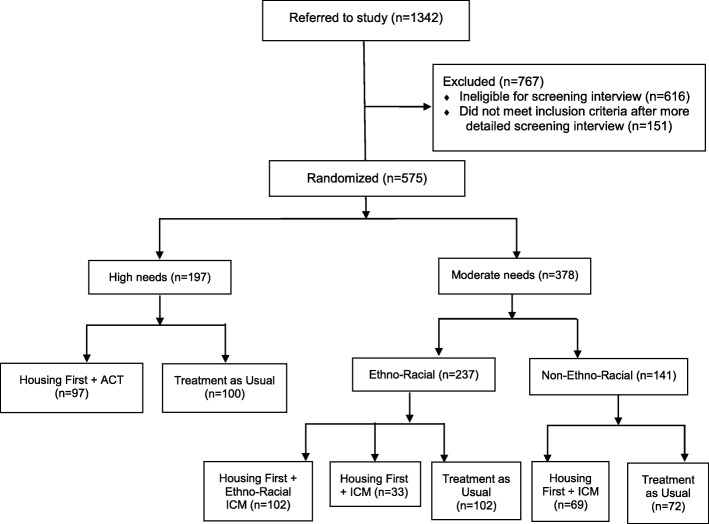
Flow of At Home/Chez Soi participants through the study in the Toronto site. *ACT* assertive community treatment, *ICM* intensive case management

According to the study protocol, as part of the Housing First model, participants received psychosocial supports geared to their level of need: ACT for high needs participants, and ICM for moderate needs participants. ACT is a mental health treatment model delivered by a multidisciplinary team (including a psychiatrist, nurse, and peer specialist) with a participant to staff ratio of 10 to 1 or less. The ACT Team met daily and provided crisis coverage at all hours. ICM is a mental health treatment model delivered primarily by a single worker with a participant to staff ratio of 20 to 1 or less. Participants were discussed at weekly case conferences and crisis coverage was available 12 h per day, 7 days per week. In Toronto, moderate needs participants who self-identified as members of a minority ethno-racial group were given a choice to participate in a regular ICM program or an ethno-racial focused ICM program, as long as space was available in both groups. We analyzed data from participants who received ACT, ICM, locally-adapted ethno-racial ICM, and treatment as usual (*n* = 575; Fig. 2).

### Ethics, consent and permissions

Individuals provided verbal consent to be screened for eligibility and to hear more about the study. Those who met inclusion criteria were assessed for capacity to consent before written informed consent was obtained from all individual participants included in the study. The study was approved by the Research Ethics Board of St. Michael’s Hospital and was prospectively registered with the International Standard Randomised Control Trial Registry (ISRCTN42520374). Full details of the study protocol have been published [25].

### Measures

Our primary measure of interest was the EQ-5D-3L, which captures 5 dimensions (mobility, self-care, usual activities, pain/discomfort, and anxiety/depression). The EQ-5D-3L measures each dimension at 3 levels (no problems, some/moderate problems, and extreme problems/unable to perform the activity), generating 243 distinct health states. We used Canadian index values to calculate community-weighted utility scores [26].

The QOLI-20 index is a quality of life measure developed for use in people with mental illness, which assesses satisfaction with family relationships, social relationships, finances, leisure, living situation, safety, and overall quality of life [17]. The QOLI-20 has demonstrated reliability and validity in people with severe mental illness, with lower scores in homeless compared with housed people with severe mental illness [27, 28]. The CSI assesses presence and frequency of mental health symptoms [20, 21], with demonstrated reliability, construct validity and responsiveness in homeless adults receiving treatment for mental illness or substance abuse [21, 29]. The RAS assesses the extent to which participants live a satisfying life within the constraints of a mental illness [18, 19]. In a recent trial of Housing First in homeless people with schizophrenia and bipolar disorder, it demonstrated satisfactory reliability and sensitivity to change, and strong correlation with the CSI and a quality of life measure [30]. Instruments were administered every 6 months except for the RAS which was administered at baseline and at 24 months.

At baseline, participants also reported which medical conditions they had experienced from a specified list of 29 health states (number of comorbidities) and answered questions about their demographic characteristics. At each 6-month visit, interviewers indicated their confidence in participants’ responses (rated as “completely confident,” “some doubts,” or “no confidence”) and assessed participants’ apparent degree of thought process impairment based on an item from the MCAS.

### Analysis and hypotheses

We characterized the study population and distribution of EQ-5D-3L index scores using descriptive statistics.

We pre-specified 4 convergent validity hypotheses. We hypothesized that the EQ-5D-3L would be strongly correlated (absolute value of the correlation coefficient *r* > 0.60 at each measurement) [31–33] with the QOLI-20, the study instrument measuring condition-specific quality of life, which we deemed to be most proximally related to generic/overall quality of life, and that with the strongest evidence for validity in our study population [27, 28]. We hypothesized that the EQ-5D-3L would be at least moderately correlated (absolute value of *r* > 0.30 at each measurement) to each of the CSI, RAS, and number of comorbidities, measuring constructs that we consider more distally related to overall quality of life. We performed Pearson’s correlation tests for each pair of measures, which rely on the assumptions that variables compared must be interval or ratio measurements, that variables are approximately normally distributed, and that there is a linear relationship between the 2 variables. Based on the assumption that data for the EQ-5D-3L and the QOLI-20, CSI, RAS, and number of comorbidities were missing at random, and according to guidelines for reporting analysis potentially affected by missing data [34], we used multiple imputation to account for missing data in our correlation calculations. We used multivariate normal regression to impute missing values of the EQ-5D-3L, QOLI-20, CSI, RAS, and number of comorbidities using 10 datasets and including the following variables in the imputation model: age, gender, ethnicity, level of need as defined by the study protocol, baseline alcohol dependence, baseline substance use dependence, treatment assignment, and time. We calculated confidence intervals using bias-corrected bootstrap confidence intervals with 500 replications for each calculation. We also used complete case analysis to perform Pearson’s correlation tests as well as Spearman’s rank correlation tests, which do not assume a normal distribution or linear relationship.

We conducted several sensitivity analyses to determine whether results were robust to different levels of mental health or thought impairment. First, given previous findings that the validity of the EQ-5D-3L may vary based on degree of impairment of mental illness, we stratified analyses according to level of need as defined by the study protocol [35]. Second, to explore whether differences in the EQ-5D-3L’s performance in people with more severe mental illness could be related to thought disorder, we stratified analyses using an item from the MCAS to classify respondents’ current level of thought process impairment (moderate to extreme vs. none to slight) [24]. Third, we restricted analyses to only those responses in which the interviewer had high confidence in participants’ responses. Finally, we restricted analyses to interviews in which the interviewer had high or moderate confidence.

We performed a linear mixed-effects repeated measure regression analysis to examine the association between EQ-5D-3L scores and QOLI-20 scores over time; this simple model accounted for the fact that measurements were repeated in the same individuals over time. We calculated the Snijders/Bosker r^2^, an established method for multilevel data [36, 37]. The funding source had no role in the study. We conducted all analyses in Stata 15.1 and used a *p*-value threshold of 0.05 for significance testing.

## Results

**Fig. 3:**
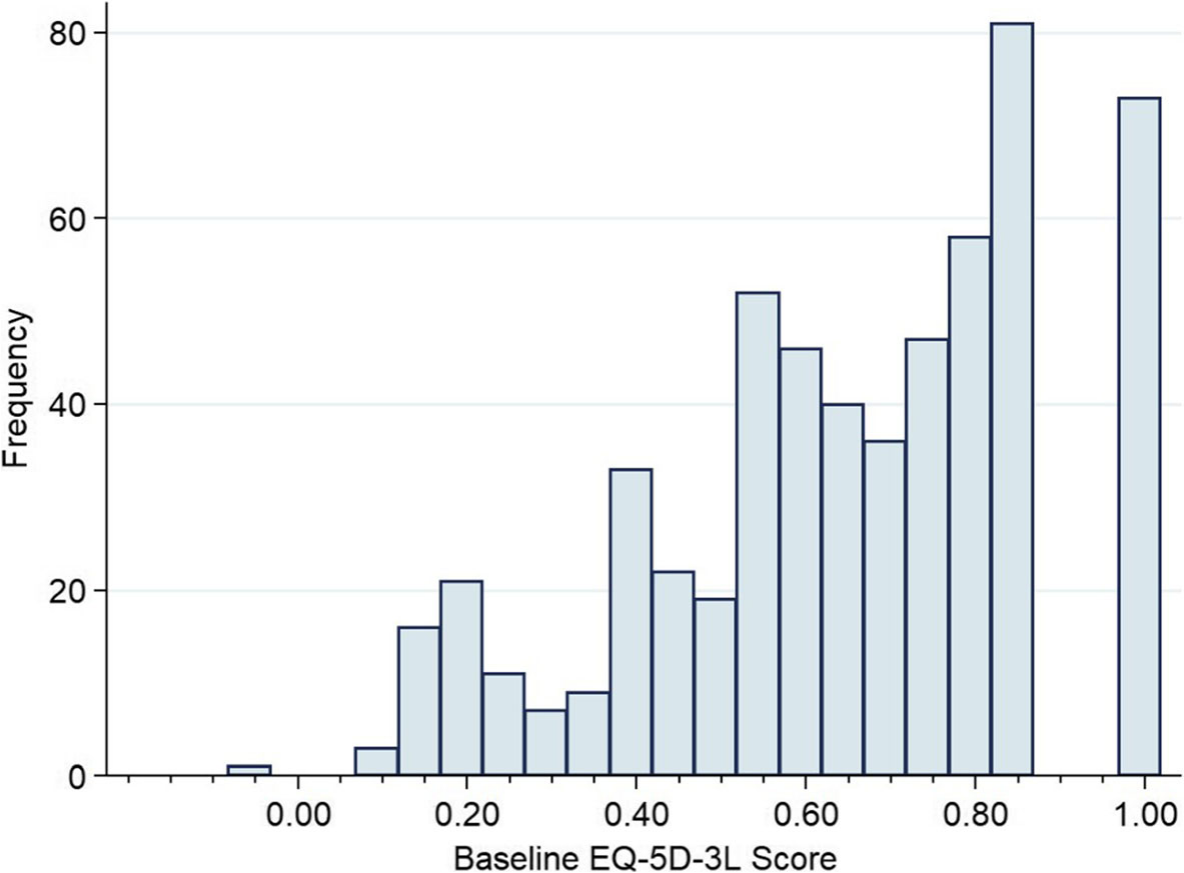
Frequency distribution of baseline EQ-5D-3L index scores

**Fig. 4:**
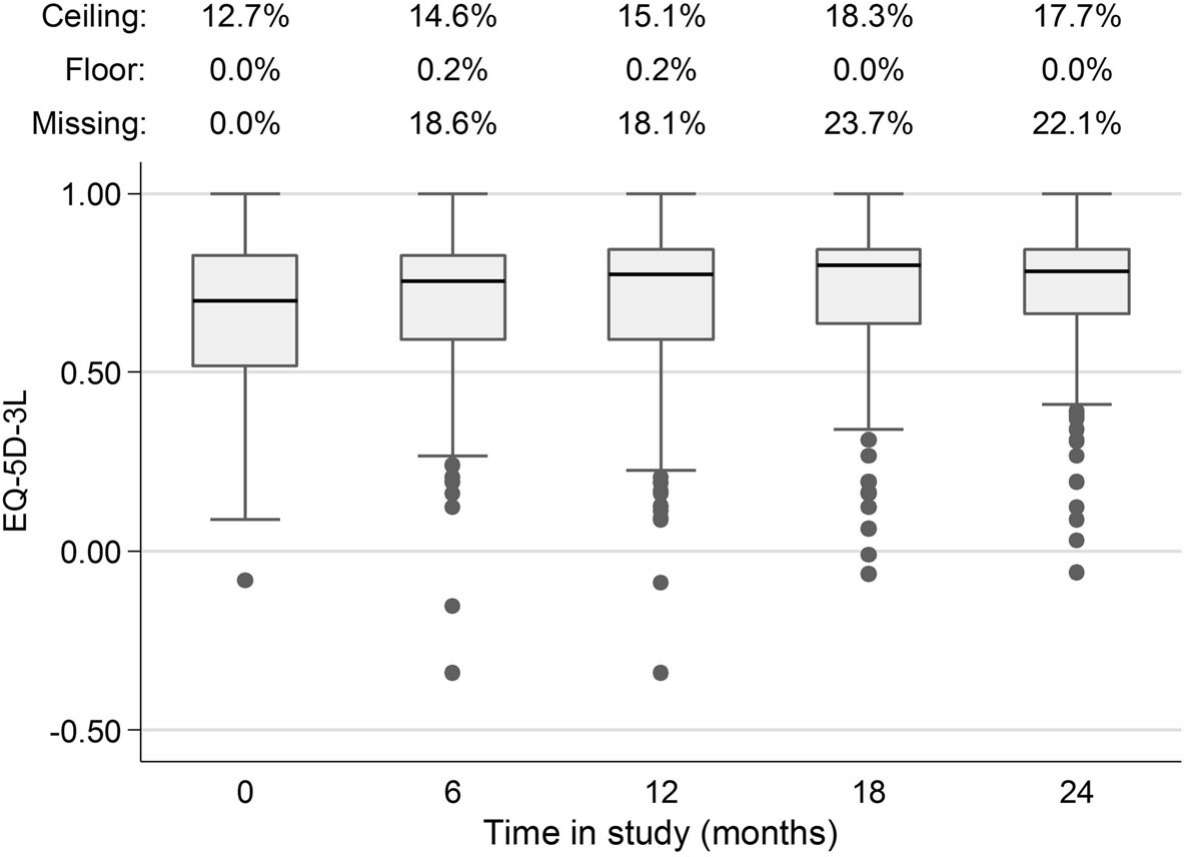
EQ-5D-3L scores over time in Toronto At Home/Chez Soi participants. This figure is a boxplot describing the distribution of EQ-5D-3L index scores at each 6 month time point in our sample. The bottom and top of the box represent the first and third quartiles of the distribution and the band inside the box is the second quartile or median. The ends of the lines or “whiskers” represent the lower quartile minus the interquartile range (IQR) and the upper quartile plus the IQR respectively

Most study participants (69%) were male, the median age was 41 years, the median lifetime duration of homelessness was 36 months, and the median longest period of homelessness was 12 months (Table 1). Only 5% of participants were employed and nearly all (95%) met criteria for “absolute” homelessness, Baseline EQ-5D-3L index scores were similar across treatment groups with an overall mean score of 0.65 and median of 0.70. The Canadian scoring algorithm does not produce scores from 0.85 to 0.99, resulting in a discontinuity in the distribution of index scores (Fig. 3). Over time, median EQ-5D-3L index scores increased, distributions narrowed, more participants reported scores at the ceiling, and the proportion of participants with missing data increased (Fig. 4). The median score for the entire sample was 0.70 (interquartile range, IQR 0.52–0.83) at baseline, 0.77 (IQR 0.59–0.84) at 12 months, and 0.78 (IQR 0.66–0.84) at 24 months. Across all time points, 15.7% of all EQ-5D-3L index scores were at the ceiling and 16.5% were missing.

**Table 1:** Baseline Characteristics of Toronto At Home/Chez Soi Participants

	High needs (*N* = 197)				Moderate needs (*N* = 378)					
	ACT (*N* = 97)		Treatment as Usual (*N* = 100)		Ethno-Racial ICM (*N* = 102)		ICM (*N* = 102)		Treatment as Usual (*N* = 174)	
	*N*	%	*N*	%	*N*	%	*N*	%	*N*	%
Age, years – median (IQR)	39 (29–47)		41 (31–50)		35 (27–48)		44 (34–47)		41 (30–40)	
Male	64	66.0	79	79.0	65	63.7	73	71.6	113	64.9
Ethnic identity^a^										
Non-ethno-racial	43	44.3	> 40	> 40	0	0	59	57.8	64	36.8
Aboriginal	7	7.1	≤5	≤5	0	0	10	9.8	8	4.6
Other ethno-racial minority	47	48.5	54	54.0	102	100	33	32.4	102	58.6
Education^b^										
Less than high school	48	49.5	53	53.5	42	41.2	61	59.8	75	43.4
Completed high school	16	16.5	22	22.2	17	16.7	17	16.7	36	20.8
Some post-secondary school	33	34.0	24	24.2	43	42.2	24	23.5	62	35.8
Homelessness (months)										
Lifetime: median (IQR)	41 (14–84)		60 (23–114)		18 (6–36)		48 (12–120)		36 (12–72)	
Longest period: median (IQR)	18 (7–48)		24 (8–60)		11 (4–18)		18 (6–54)		12 (5–36)	
Thought impairment	78	80.4	75	75	29	28.4	15	14.7	32	18.9
Baseline EQ-5D score										
Mean ± SD	0.67 ± 0.26		0.67 ± 0.26		0.64 ± 0.23		0.62 ± 0.22		0.66 ± 0.21	
Median (IQR)	0.73(0.49–0.83)		0.71(0.46–0.83)		0.70(0.52–0.83)		0.66 (0.49–0.78)		0.67(0.54–0.83)	
Baseline EQ-5D score at floor	0	0	0	0	0	0	0	0	0	0
Baseline EQ-5D score at ceiling	18	18.6	22	22.0	10	9.8	7	6.9	16	9.2

*ACT* assertive community treatment, *ICM* intensive case management, *IQR* interquartile range, *SD* standard deviation

^a^ The distribution of ethnic identity is suppressed to prevent small cells (N ≤ 5) and preserve confidentiality

^b^ Education data were missing for 2 participants

**Fig. 5:**
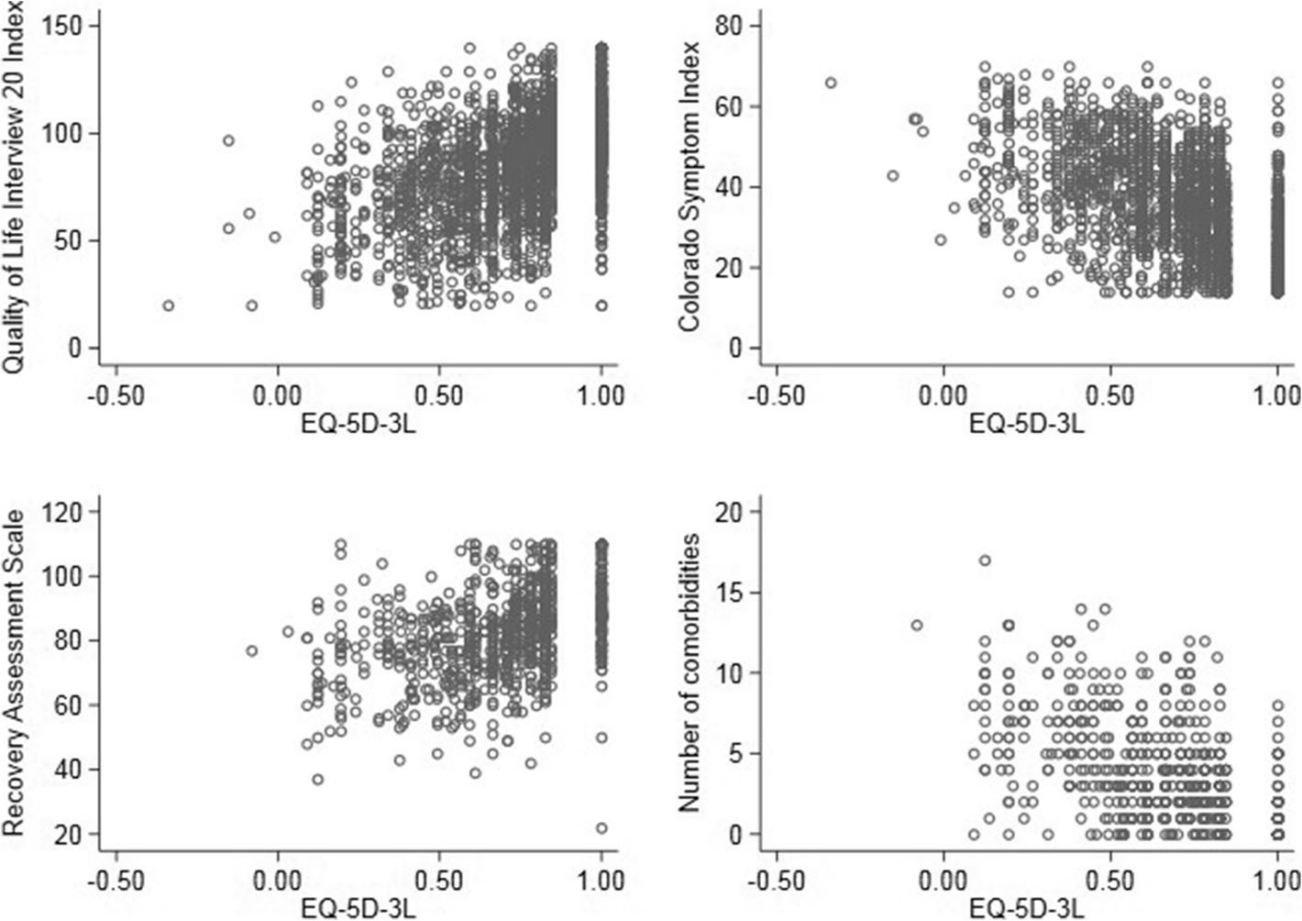
Scatterplots of EQ-5D-3L index scores and other outcome measures at baseline

In correlation calculations using multiple imputation, the EQ-5D-3L did not meet our pre-specified hypothesis of strong correlation (|r| > 0.60) with the QOLI-20 at any time point (Table 2 and Fig. 5). The EQ-5D-3L was moderately correlated (|r| > 0.3) with the CSI and RAS at all time points and with the number of medical comorbidities at baseline, meeting our pre-specified hypotheses for tests of validity.

**Table 2:** Correlations between EQ-5D-3L and Other Outcome Measures Using Multiple Imputation

Stratum and Time	QOLI-20	CSI	RAS	Comorbidities
All				
0	0.52 (0.47 to 0.58)	−0.57^a^ (− 0.63 to − 0.51)	0.42^a^ (0.36 to 0.47)	− 0.51^a^ (− 0.57 to − 0.44)
6	0.43 (0.37 to 0.48)	− 0.53^a^ (− 0.58 to − 0.48)	–	–
12	0.47 (0.41 to 0.53)	− 0.54^a^ (− 0.59 to − 0.48)	–	–
18	0.40 (0.33 to 0.45)	− 0.47^a^ (− 0.54 to − 0.42)	–	–
24	0.37 (0.31 to 0.43)	− 0.47^a^ (− 0.52 to − 0.41)	0.31^a^ (0.24 to 0.38)	–
High Needs Mental Illness				
0	0.49 (0.38 to 0.58)	− 0.63^a^ (− 0.71 to − 0.52)	0.43^a^ (0.34 to 0.52)	− 0.59^a^ (− 0.68 to − 0.49)
6	0.40 (0.32 to 0.49)	− 0.51^a^ (− 0.60 to − 0.43)	–	–
12	0.46 (0.39 to 0.52)	− 0.52^a^ (− 0.58 to − 0.43)	–	–
18	0.42 (0.31 to 0.52)	− 0.51^a^ (− 0.59 to − 0.42)	–	–
24	0.39 (0.28 to 0.48)	− 0.50^a^ (− 0.58 to − 0.40)	0.34^a^ (0.22 to 0.44)	–
Moderate Needs Mental Illness				
0	0.54 (0.46 to 0.59)	− 0.53^a^ (− 0.58 to − 0.45)	0.40^a^ (0.33 to 0.49)	−0.44^a^ (− 0.54 to − 0.36)
6	0.44 (0.38 to 0.52)	−0.55^a^ (− 0.62 to − 0.48)	–	–
12	0.48 (0.40 to 0.55)	−0.55^a^ (− 0.62 to − 0.48)	–	–
18	0.38 (0.30 to 0.46)	−0.45^a^ (− 0.53 to − 0.37)	–	–
24	0.35 (0.27 to 0.43)	−0.45^a^ (− 0.51 to − 0.37)	0.30 (0.21 to 0.37)	–
High Thought Impairment				
0	0.52 (0.42 to 0.61)	−0.62^a^ (− 0.71 to − 0.53)	0.40^a^ (0.30 to 0.49)	−0.56^a^ (− 0.65 to − 0.47)
6	0.35 (0.21 to 0.45)	−0.55^a^ (− 0.64 to − 0.44)	–	–
12	0.44 (0.32 to 0.54)	−0.59^a^ (− 0.67 to − 0.49)	–	–
18	0.41 (0.22 to 0.53)	−0.48^a^ (− 0.62 to − 0.27)	–	–
24	0.41 (0.27 to 0.55)	−0.45^a^ (− 0.59 to − 0.24)	0.24 (− 0.02 to 0.41)	–
Low Thought Impairment				
0	0.52 (0.44 to 0.58)	− 0.52^a^ (− 0.59 to − 0.45)	0.43^a^ (0.35 to 0.51)	−0.45^a^ (− 0.55 to − 0.35)
6	0.47 (0.37 to 0.54)	−0.54^a^ (− 0.62 to − 0.47)	–	–
12	0.49 (0.40 to 0.57)	−0.52^a^ (− 0.60 to − 0.42)	–	–
18	0.38 (0.28 to 0.48)	−0.46^a^ (− 0.54 to − 0.37)	–	–
24	0.33 (0.23 to 0.43)	−0.45^a^ (− 0.52 to − 0.37)	0.33^a^ (0.24 to 0.42)	–
High Interview Confidence Only				
0	0.54 (0.45 to 0.61)	−0.48^a^ (− 0.57 to − 0.33)	0.39^a^ (0.28 to 0.51)	−0.45^a^ (− 0.56 to − 0.33)
6	0.49 (0.39 to 0.57)	−0.53^a^ (− 0.61 to − 0.41)	–	–
12	0.51 (0.38 to 0.61)	−0.46^a^ (− 0.58 to − 0.36)	–	–
18	0.38 (0.25 to 0.49)	−0.48^a^ (− 0.58 to − 0.35)	–	–
24	0.42 (0.30 to 0.52)	−0.53^a^ (− 0.62 to − 0.41)	0.37^a^ (0.24 to 0.46)	–
High or Moderate Interviewer Confidence Only				
0	0.52 (0.46 to 0.57)	−0.56^a^ (− 0.62 to − 0.51)	0.42^a^ (0.35 to 0.48)	−0.49^a^ (− 0.56 to − 0.42)
6	0.40 (0.34 to 0.47)	−0.52^a^ (− 0.57 to − 0.45)	–	–
12	0.47 (0.40 to 0.55)	−0.52^a^ (− 0.59 to − 0.45)	–	–
18	0.38 (0.31 to 0.46)	−0.45^a^ (− 0.52 to − 0.37)	–	–
24	0.35 (0.27 to 0.44)	−0.45^a^ (− 0.52 to − 0.38)	0.34^a^ (0.28 to 0.42)	–

*QOLI-20* Lehman Quality of Life Interview 20 index total, *CSI* Colorado Symptom Index, *RAS* Recovery Assessment Scale

^a^ Met pre-defined threshold for correlation (*r* > |0.6| for QOLI-20, *r* > |0.3| for CSI, RAS, and number of comorbidities)

Results were similar when we stratified analyses by level of need or degree of thought impairment or when we restricted analyses to only those instances in which the interviewer confidence in responses was at least high or at least moderate (Table 2). Similarly, analyzing the data using complete case analysis and a non-parametric test did not meaningfully change the results (Additional file 1). Using complete case analysis, without multiple imputation to account for missing data, the EQ-5D-3L no longer surpassed the threshold for moderate correlation with the RAS at 24 months.

The Snijders/Bosker r^2^ for longitudinal validity between the EQ-5D-3L and QOLI-20 within subjects was 0.2094, indicating the proportion of variance in QOLI-20 scores for individuals explained by variance in EQ-5D-3L scores over time. The square root of this value, 0.4576, is analogous to the correlation coefficients, r, in the cross-sectional analyses.

## Discussion

In a study of Housing First for homeless adults with mental illness, the EQ-5D-3L was moderately correlated with scores from the QOLI-20 at each study time point and in the longitudinal analysis. This finding did not support our pre-specified hypothesis that there would be a strong correlation with the QOLI-20, a condition-specific quality of life instrument that has been extensively evaluated. Nevertheless, we found that the EQ-5D-3L met 3 tests of convergent validity, demonstrating moderate correlation with the presence and frequency of mental health symptoms, recovery as measured by the RAS, and number of medical comorbidities.

There are three leading explanations for our finding that the EQ-5D-3L did not strongly correlate with the QOLI-20 in this sample. First, we observed large variation in baseline EQ-5D-3L index scores. Random measurement error is well-known to attenuate the correlation coefficient between two variables [38]. Error could be introduced in this sample if participants did not answer the EQ-5D-3L questionnaire accurately due to symptoms of their mental illness. Indeed, in previous studies, the content validity of the EQ-5D-3L compared with disorder-specific scales was lower among people with schizophrenia than among those with other mental disorders [35]. Thought impairment, a symptom of schizophrenia and other severe mental illness as well as intoxication, may be one cause of inaccurate reporting; importantly, 78% of high needs and 20% of moderate needs participants in our sample demonstrated thought impairment. Furthermore, 72% of the 1500 participants in the full At Home/Chez Soi sample who completed neuropsychological assessments demonstrated some degree of cognitive impairment [39]. However, sensitivity analyses in which we restricted our evaluations to individuals with lower degree of thought impairment or to those responses in which interviewers had high confidence did not significantly change our results, making it less likely that measurement error related to inaccurate reporting secondary to thought impairment fully explains our findings.

A second explanation for our findings is that the EQ-5D-3L and QOLI-20 are measuring different domains of quality of life. The EQ-5D-3L was developed as a generic utility instrument to be used across health states. Thus, its domains are general and include mobility, self-care, usual activities, pain and discomfort, and depression and anxiety; perhaps not surprisingly, we found that the correlation between the EQ-5D-3L and number of medical comorbidities exceeded our threshold for moderate correlation. However, qualitative research studies indicate that the domains of the EQ-5D-3L have only modest overlap with domains of quality of life identified as important by people with mental health problems [10, 40]. The domains included in the QOLI-20 (such as satisfaction with living situation and finances) may be more relevant for people who are homeless and living with mental illness. Given the importance of effectively measuring health utility for cost effectiveness analysis in this population, it is critical to determine whether other indirect utility instruments with a larger number of domains, such as the Health Utilities Index [41, 42], have better measurement properties than the EQ-5D-3L.

Finally, the levels included in the EQ-5D-3L may not be sufficiently discriminative to distinguish important quality of life effects. At any time, fewer than 4% of participants endorsed the most severe (third) level of the domains related to mobility (“I am confined to bed”), self-care (“I am unable to wash or dress myself”), or usual activities (“I am unable to perform my usual activities”). These skewed distributions may lead to problems with discrimination for participants with varying degrees of difficulty in these dimensions who would all be grouped into the second level in the current response scale. Furthermore, the summary score of the EQ-5D-3L has a discontinuity at the upper range, further limiting its ability to discriminate between individuals at the high end of the scale. A newer version of the EQ-5D, released after our study was initiated, incorporates 5 levels for each domain, does not have a discontinuity and is less prone to ceiling effects [43–45].

Our study has some limitations. We had a large proportion of missing data, as high as 23.7% at 18 months, and it is possible that participants with poorer quality of life may have been more likely to be lost to follow-up (and that missing EQ-5D-3L scores would have been systematically lower). However, convergent validity between the EQ-5D-3L and other instruments was not better at baseline, when data were complete, and did not change significantly when analyzed using multiple imputation [34, 46]. Furthermore, the linear mixed effects model is robust to missing data under a missing at random assumption [47, 48]. Second, although all of the interviewers were trained, they were not blinded to treatment assignment and we were not able to control for possible individual interviewer effects in our analyses. Finally, we used data only from homeless adults with mental illness in Toronto, a large urban centre in Canada. Our findings may not be generalizable to other settings.

## Conclusions

We know of no other study that has assessed the convergent validity of an indirect utility instrument in homeless adults with mental illness. We found only moderate support for the EQ-5D-3L, suggesting that further psychometric testing, including with the EQ-5D-5L, is warranted to determine the optimal method for producing utility scores for this population. We urge caution when using the EQ-5D-3L as a measure of quality of life and also for the closely related concept of health utility, including in cost-utility analyses of health and social interventions for adults with severe mental illness and unstable social situations. A finding of no difference in utility scores may be due to limitations of the instrument rather than lack of effectiveness of the intervention. Until additional research is available, economic analyses should include a broad range of outcomes in sensitivity analyses.

## Additional file

Additional file 1: Correlations between EQ-5D-3L and Other Outcome Measures Using Complete Case Analysis. (DOCX 16 kb)

## Data Availability

The At Home/Chez Soi project has a process by which interested investigators who would like to use the data for publication can make a formal request. The formal request is reviewed by a cross-site committee and as long as those particular analyses have not already been undertaken approval and data sharing can take place.

## Abbreviations

ACT: Assertive community treatment
CSI: Colorado Symptom Index
EQ-5D-3L: EuroQol-5 Dimension 3-level questionnaire
ICM: Intensive case management
IQR: Interquartile range
MCAS: Multnomah Community Ability Scale
MINI: Mini International Neuropsychiatric Interview
NICE: National Institute for Health and Care Excellence
QOLI-20: Lehman Quality of Life Interview 20
RAS: Recovery Assessment Scale
SF-36: 36-Item Short Form Survey
