# Supplementary material for: Convergent validity of the EQ-5D-3L in a randomized-controlled trial of the Housing First model
Source: BMC Health Serv Res. 2019 Jul 12;19:482. doi: 10.1186/s12913-019-4310-z (PMC6626335; doi:10.1186/s12913-019-4310-z)
Supplement: Supplementary file 1 — Correlations between EQ-5D-3L and Other Outcome Measures Using Complete Case Analysis. (DOCX 16 kb) [file 12913_2019_4310_MOESM1_ESM.docx]

**Additional File 1.** Correlations between EQ-5D-3L and Other Outcome Measures Using Complete Case Analysis^1^

| Stratum and Time | | QOLI-20 | | CSI | | RAS | | Comorbidities |
| --- | --- | --- | --- | --- | --- | --- | --- | --- |
| All | |  | |  | |  | |  |
| 0 | | 0.52 (0.45 to 0.58); n=522 | | -0.57* (-0.62 to -0.51); n=526 | | 0.41* (0.33 to 0.48); n=468 | | -0.51* (-0.56 to -0.44); n=575 |
| 6 | | 0.40 (0.32 to 0.48); n=416 | | -0.53* (-0.60 to -0.46); n=421 | | - | | - |
| 12 | | 0.49 (0.41 to 0.56); n=419 | | -0.54* (-0.61 to -0.47); n=429 | | - | | - |
| 18 | | 0.38 (0.29 to 0.46); n=386 | | -0.47* (-0.54 to -0.39); n=400 | | - | | - |
| 24 | | 0.31 (0.22 to 0.40); n=398 | | -0.43* (-0.50 to -0.35); n=415 | | 0.30 (0.20 to 0.39); n=374 | | - |
| High Needs Mental Illness | | | |  | |  | |  |
| 0 | | 0.46 (0.34 to 0.57); n=170 | | -0.62* (-0.70 to -0.52); n=174 | | 0.42* (0.28 to 0.55); n=147 | | -0.59* (-0.68 to -0.49); n=197 |
| 6 | | 0.37 (0.21 to 0.50); n=138 | | -0.51* (-0.62 to -0.37); n=137 | | - | | - |
| 12 | | 0.48 (0.34 to 0.60); n=136 | | -0.54* (-0.65 to -0.40); n=129 | | - | | - |
| 18 | | 0.38 (0.22 to 0.52); n=123 | | -0.52* (-0.64 to -0.38); n=125 | | - | | - |
| 24 | | 0.29 (0.13 to 0.45); n=126 | | -0.45* (-0.57 to -0.30); n=135 | | 0.32* (0.15 to 0.47); n=122 | | - |
| Moderate Needs Mental Illness | | | | | |  | |  |
| 0 | | 0.55 (0.47 to 0.62); n=352 | | -0.53* (-0.60 to -0.46); n=352 | | 0.39* (0.30 to 0.48); n=321 | | -0.44* (-0.52 to -0.36); n=378 |
| 6 | | 0.42 (0.31 to 0.51); n=278 | | -0.55* (-0.63 to -0.46); n=284 | | - | | - |
| 12 | | 0.49 (0.39 to 0.57); n=283 | | -0.55* (-0.63 to -0.47); n=300 | | - | | - |
| 18 | | 0.37 (0.26 to 0.47); n=263 | | -0.44* (-0.53 to -0.34); n=275 | | - | | - |
| 24 | | 0.31 (0.20 to 0.41); n=272 | | -0.42* (-0.51 to -0.32); n=280 | | 0.29 (0.17 to 0.40); n=252 | | - |
| High Thought Impairment | | | |  | |  | |  |
| 0 | | 0.51 (0.40 to 0.61); n=203 | | -0.62* (-0.70 to -0.53); n=205 | | 0.38* (0.24 to 0.50); n=179 | | -0.56* (-0.65 to -0.47); n=229 |
| 6 | | 0.31 (0.12 to 0.47); n=108 | | -0.55* (-0.67 to -0.41); n=114 | | - | | - |
| 12 | | 0.46 (0.31 to 0.60); n=115 | | -0.61* (-0.71 to -0.49); n=118 | | - | | - |
| 18 | | 0.41 (0.22 to 0.57); n=87 | | -0.48* (-0.62 to -0.30); n=89 | | - | | - |
| 24 | | 0.35 (0.13 to 0.54); n=72 | | -0.43* (-0.60 to -0.23); n=78 | | 0.23 (-0.01 to 0.44); n=68 | | - |
| Low Thought Impairment | | | |  | |  | |  |
| 0 | | 0.52 (0.43 to 0.59); n=319 | | -0.52* (-0.60 to -0.44); n=321 | | 0.43* (0.33 to 0.52); n=289 | | -0.45* (-0.53 to -0.37); n=346 |
| 6 | | 0.44 (0.35 to 0.53); n=307 | | -0.53* (-0.60 to -0.44); n=306 | | - | | - |
| 12 | | 0.49 (0.40 to 0.58); n=304 | | -0.52* (-0.60 to -0.44); n=311 | | - | | - |
| 18 | | 0.36 (0.26 to 0.46); n=298 | | -0.47* (-0.55 to -0.38); n=310 | | - | | - |
| 24 | | 0.30 (0.20 to 0.40); n=325 | | -0.44* (-0.52 to -0.34); n=336 | | 0.32* (0.21 to 0.42); n=305 | | - |
| High Interviewer Confidence Interviews Only | | | | | | | |  |
| 0 | | 0.55 (0.45 to 0.64); n=193 | | -0.49* (-0.59 to -0.37); n=195 | | 0.40* (0.26 to 0.52); n=165 | | -0.45* (-0.55 to -0.33); n=204 |
| 6 | | 0.50 (0.37 to 0.61); n=155 | | -0.54* (-0.64 to -0.42); n=159 | | - | | - |
| 12 | | 0.51 (0.40 to 0.61); n=179 | | -0.44* (-0.55 to -0.31); n=184 | | - | | - |
| 18 | | 0.38 (0.24 to 0.51); n=165 | | -0.49* (-0.60 to -0.37); n=172 | | - | | - |
| 24 | | 0.40 (0.26 to 0.52); n=164 | | -0.51* (-0.61 to -0.39); n=175 | | 0.35* (0.20 to 0.48); n=160 | | - |
| High or Moderate Interviewer Confidence Interviews Only | | | | | | | |  |
| 0 | | 0.51 (0.45 to 0.58); n=502 | | -0.56* (-0.62 to -0.50); n=505 | | 0.42* (0.34 to 0.50); n=454 | | -0.49* (-0.55 to -0.43); n=550 |
| 6 | | 0.39 (0.31 to 0.47); n=411 | | -0.52* (-0.59 to -0.45); n=412 | | - | | - |
| 12 | | 0.49 (0.41 to 0.56); n=404 | | -0.53* (-0.60 to -0.46); n=414 | | - | | - |
| 18 | | 0.37 (0.28 to 0.45); n=381 | | -0.45* (-0.53 to -0.37); n=393 | | - | | - |
| 24 | | 0.32 (0.23 to 0.40); n=394 | | -0.44* (-0.51 to -0.35); n=411 | | 0.33* (0.24 to 0.42); n=370 | | - |
| All, non-parametric | | | |  | |  | |  |
| 0 | 0.53 (0.46 to 0.59); n=522 | | -0.58* (-0.63 to -0.52); n=526 | | 0.42* (0.35 to 0.50); n=468 | | -0.50* (-0.56 to -0.43); n=575 | |
| 6 | 0.42 (0.34 to 0.50); n=416 | | -0.55* (-0.61 to -0.48); n=421 | | - | | - | |
| 12 | 0.47 (0.39 to 0.54); n=419 | | -0.56* (-0.62 to -0.49); n=429 | | - | | - | |
| 18 | 0.39 (0.30 to 0.47); n=386 | | -0.48* (-0.55 to -0.40); n=400 | | - | | - | |
| 24 | 0.32 (0.23 to 0.41); n=398 | | -0.46* (-0.53 to -0.38); n=415 | | 0.35* (0.26 to 0.44); n=374 | | - | |

QOLI-20 Lehman Quality of Life Interview 20 index total, CSI Colorado Symptom Index, RAS Recovery Assessment Scale.

*Met pre-defined threshold for correlation (r>|0.6| for QOLI-20, r>|0.3| for CSI, RAS, and number of comorbidities)

^1^ indicates strength of correlation using Pearson’s test unless otherwise indicated
